# The implementation and adherence to evidence-based protocols for psychotherapy for depression: the perspective of therapists in Dutch specialized mental healthcare

**DOI:** 10.1186/s12888-018-1768-x

**Published:** 2018-06-14

**Authors:** Sanne J. E. Bruijniks, Gerdien Franx, Marcus J. H. Huibers

**Affiliations:** 1 Department of Clinical Psychology, VU Amsterdam, Van der Boechorststraat 1, 1081 BT Amsterdam, the Netherlands; 2 113 Zelfmoordpreventie, Amsterdam, the Netherlands; 3 Department of Psychology, University of Pennsylvania, Philadelphia, USA

**Keywords:** Cognitive-behavioral therapy, Interpersonal psychotherapy, Psychotherapy, Implementation, Protocol adherence, Specialized mental healthcare, Depression

## Abstract

**Background:**

Although psychotherapy is an effective treatment for depression, a large number of patients still do not receive care according to the protocols that are used in clinical trials. Instead, patients often receive a modified version of the original intervention. It is not clear how and when treatment protocols are used or modified in the Dutch specialized mental health care and whether these changes lead to suboptimal adherence to treatment protocols.

**Methods:**

In the context of an ongoing multicenter trial that investigates whether twice-weekly sessions of protocolized interpersonal psychotherapy (IPT) and cognitive behavioral therapy (CBT) for depression lead to better treatment outcomes compared to once-weekly sessions, two focus groups using semi-structured interviews were organized. Aims were to increase insight in the adherence to and modifications of CBT and IPT protocols in the Dutch specialized mental health care for depression. Participants were fifteen therapists from seven mental health locations part of five mental health organizations. Verbatim transcripts were coded and analyzed using qualitative software.

**Results:**

Three themes emerged: modification as the common practice, professional and patient factors influencing the adherence to protocols and organizational boundaries and flexibility. Treatment modification appeared to happen on a frequent basis, even in the context of a trial. Definitions of treatment modifications were multiple and varied from using intuition to flexible use of the same protocol. Therapist training and supervision, the years of work experience and individual characteristics of the therapist and the patient were mentioned to influence the adherence to protocols. Modifications of the therapists depended very much on the culture within the mental health locations, who differed in terms of the flexibility offered to therapists to choose and modify treatment protocols.

**Conclusions:**

Not all treatment modifications were in line with existing evidence or guidelines. Regular supervision, team meetings and a shared vision were identified as crucial factors to increase adherence to treatment protocols, whereas additional organizational factors, among which a change of mindset, may facilitate adequate implementation.

## Background

Adequate implementation of protocolized, evidence-based psychotherapy such as cognitive behavioral therapy (CBT) and interpersonal psychotherapy (IPT) has been associated with better treatment outcomes [1, 2]. Unfortunately, a large number of patients still receives inadequate or suboptimal psychological treatment [3–9]. Although CBT and IPT are the most investigated psychotherapies for depression [10, 11] and among the first-choice treatments for different levels of severity and subtypes of depression [12], therapists have been observed to drift from treatment protocols and modify them in different ways. For example, therapists may drift from treatment protocols by focusing on an immediate stressor, rushing into third-wave therapies before offering the first-choice of treatment or by leaving elements out [7, 13]. Also, it was shown that therapists may modify treatment protocols on the basis of their own, or patient preferences, even when these are inconsistent with evidence-based treatment guidelines [9, 14].

Not all modifications of treatment protocols might lead to suboptimal treatment or worse treatment outcomes. A systematic review on the use of various preventive, promoting or treatment interventions in mental health care pointed to twelve different ways in which a protocol can be modified and defined treatment fidelity as the adherence to the intervention components, competence with which the intervention is delivered and the differentiation from other treatments. In addition, the authors distinguish between fidelity-consistent modifications that do not alter core elements of treatment enough to reduce adherence to a protocol and do not reduce ability to differentiate between treatments, and fidelity-inconsistent modifications, such as modifications that reduce or preclude the delivery of core elements or decrease ability to differentiate between treatments [15, 16]. Removing treatment elements, losing structure, drifting from protocol or using inconsistent treatment strategies were framed as fidelity-inconsistent, and changing the length of the intervention or adding consistent modules were hypothesized as fidelity-consistent. The study of Levitz and colleagues provides a good example of modifications that seem to be in line with the protocol. In this study, flexible use of the CBT protocol for posttraumatic stress disorder (PTSD) was defined as skipping protocol sessions if the therapist did not deem the material appropriate for the patient, repeating certain protocol sessions if the material was particularly relevant to the given patient, ending therapy with a patient before completing all 16 sessions or having non- protocol sessions in order to discuss a current life stressor that required significant session time. Although patients in the flexible protocol group received on average three more sessions, of which one non-protocol session, using the protocol in a flexible way led to the same results as strict adherence to the protocol in this randomized controlled trial (RCT) [17]. Also, other studies found no evidence that small modifications such as refining the protocol or using protocols in a flexible way may lead to diminished effects of treatment outcomes and have even related treatment flexibility to more engagement in CBT for children with anxiety disorders [18, 19].

Information about how treatment protocols are used and modified in Dutch clinical practice is limited. Yet serious concerns about the quality of CBT in the Netherlands have been raised: it was suggested that limited educational programs, the tendency to attend to new interventions that are popular, but less evidence-based and limitations within the organization, such as insufficient supervision or high workload, may hinder adequate implementation of CBT [20]. Also, one of the possible reasons for why a recent observational study showed that supportive therapy was the most registered treatment among patients with a major depressive disorder in the Netherlands [21], is that therapists combined elements of CBT with elements from other therapies and therefore registered the treatment as supportive therapy [21].

In the Netherlands, a RCT is currently conducted [22] into the effectiveness of a higher session frequency of protocolized CBT and IPT for depression in specialized mental health care. CBT for depression is based on the manual by Beck [23], whereas IPT is based on the manual by Klerman [24]. The goal of the trial is to investigate whether twice-weekly sessions will lead to better treatment outcomes compared to once-weekly sessions. This trial was considered to be a good context to generate more in-depth knowledge of adherence to and modifications of protocols for IPT and CBT for depression by therapists from different mental health locations and areas in the Netherlands.

The aim of the present qualitative study was to learn about the perspective of therapists of providing CBT or IPT treatment according to protocols in daily practice. Insight in how treatment protocols are used or modified by clinicians might help to design strategies to improve the implementation and delivery of CBT and IPT in specialized mental health care the Netherlands.

## Method

### Study design

The study used a qualitative approach by conducting two focus groups with therapists from specialized mental health organizations that participated in a randomized trial in the Netherlands, called the FreqMech study [22]. In the FreqMech study, 200 patients were randomized into: a) 16 twice-weekly CBT sessions followed by 4 sessions during the last 8 weeks, b) 16 twice-weekly IPT sessions followed by 4 sessions during the last 8 weeks, c) 16 once-weekly CBT sessions followed by 4 sessions during the last 8 weeks, d) 16 once-weekly IPT sessions followed by 4 sessions during the last 8 weeks. All patients receive 12 to 20 sessions, 45 min in length of protocolized IPT [24] or CBT [23], depending on the individual progress of the patient. Both treatments are time-limited and target the present symptoms. However, the treatments have a different focus: key elements of the CBT protocol are behavioral activation and challenging dysfunctional beliefs (i.e. negative beliefs about the self, other or the world), whereas the IPT protocol focuses on the interpersonal context of the depressive symptoms. The focus groups were conducted in parallel with the data collection of the trial, which provided a good context to study the perspective of professionals on the adherence to treatment protocols.

### Data collection and participants

A representative selection of therapists from nine locations of six mental health organizations that had been, or were currently involved in the FreqMech study, were invited by a formal letter from the research group to participate in a two-hour focus group session. Selection criteria of the therapists are given in Table 1.

**Table 1:** Therapist selection criteria

• Mental health center	
• Professional background	
• Years of work experience	
• Years of work experience with CBT and/or IPT	

For each mental health location, initially 3–5 therapists with different professions and different levels of experience, were invited. In case of no response, additional therapists in the same mental health location were invited. This selection procedure resulted in a total of forty-six therapists that were invited, out of a total of 70 therapists that had been involved in the Freqmech trial until May 2017. The invitations resulted in two focus groups with six and nine participants respectively from seven different mental health locations in five different mental health care organizations. We were not able to recruit therapists from two of the nine mental health locations participating in the FreqMech study, due to a lack of time expressed by the therapists to participate in the focus groups. Therapists represented different professions, different levels of experience, different types of organizations (academic versus non-academic) and worked in different areas of the Netherlands. The sample seemed a representative selection of the therapists in the FreqMech study, were the largest profession groups were: licensed mental health psychologists (30.88%), psychologists MSc (25%), psychologists MSc in training for licensed mental health psychologists (13.23%) or psychotherapists (7.35%). Characteristics of the participants in the present and the FreqMech study (i.e. until May 2017) can be found in Table 2.

**Table 2:** Demographics, professions and years of clinical experience of the participants

	Focus group 1 (*n* = 6)	Focus group 2 (*n* = 9)	FreqMech therapist sample (*n* = 70)
Demographics			
Female (%)	83.4%	100%	81.4%
Age (years + range)	36.80 (26.61–55.25)	40.63 (27.91–58.49)	39.02 (25–60)
Profession (n)^a^			
Clinical Nurse Specialist	1 (16.66%)	0	4 (5.71%)
Clinical Psychologist	0	0	2 (2.85%)
Licensed mental health psychologist (GZ-psycholoog) in training for clinical psychologist	1 (16.66%)	1 (11.11%)	3 (4.28%)
Licensed mental health psychologist (GZ-psycholoog) & psychotherapist	0	0	3 (4.28%)
Licensed mental health psychologist (GZ-psycholoog)	2 (33.33%)	2 (22.22%)	22 (30.88%)
Psychologist MSc in training for licensed mental health psychologist	1 (16.66%)	4 (44.44%)	9 (13.23%)
Psychologist MSc	1 (16.66%)	0	18 (25%)
Psychotherapist	0	1 (11.11%)	5 (7.35%)
Psychotherapist in training	0	0	2 (2.85%)
Psychiatrist	0	1 (11.11%)	2 (2.85%)
Clinical experience (in years)			
General (median + range)	7.75 (10; 2–14)	11.22 (8; 5–28)	10.02 (7; 1–29)
CBT (median + range)	5.83 (4; 2–12)	7.11 (6; 0–28)	7.20 (5; 1–26)
IPT (median + range)	1.75 (1; 0–3)	3.66 (1; 0–22)	3.25 (.5; 0–25)

^a^Professions according to the Dutch educational system; psychologist MSc refers to psychological training on master level, a clinical nurse specialist is a nurse with three additional years of postgraduate training in mental health care and licensed to provide CBT and IPT treatment; licensed mental health psychologists, psychotherapists, and clinical psychologists received two, three and six additional years of postgraduate training, respectively. For FreqMech sample, years of therapist experience at the start of participating on the FreqMech study was noted. In addition, some data was missing on the age and years of experience variables, available data per variable was: age (*n* = 60), years of experience (*n* = 58), CBT experience (*n* = 54), IPT experience (*n* = 37)

The focus groups were held at the Vrije Universiteit Amsterdam and were facilitated by a moderator (GF) and assistant moderator (SB). We used a semi-structured interview guide for both focus groups, developed by the researchers. All researchers were involved and played a different role in the development of the semi-structured interview guide. MH was involved as a professor in Clinical Psychology and a psychotherapist working in one of the mental health care organizations, GF was involved as an expert on implementation of protocols and was not involved in the data collection of the FreqMech study, and SB was involved as a researcher (PhD student) who conducted the data collection for the FreqMech study. The interview guide consisted of questions related to: (1) adherence to treatment protocols, (2) which type of modifications are applied in daily practice, (3) which patient and therapist characteristics influence the modification of the protocols chosen by the therapists and, (4) in what way the organizational context influenced the way therapists adhere to protocols. Each focus group discussion started with an introduction in which the moderators and the participants presented themselves and in which the moderators the purpose of the study was explained. The rationale of the study that was told to the therapists was that we encountered an increasing use of terms such as ‘personalizing treatments’ during the data collection of the FreqMech study and that this led to the research questions we identified in our interview guide. During the second focus group, in order to gain more in-depth information and prevent a repetition of responses, the moderator and assistant moderator briefly summarized the content of the first focus group and asked the group to verify, add to and elaborate on the issues raised during the first group.

### Data analyses

Focus groups were videotaped and transcribed verbatim by an independent research assistant. Participants were given anonymous identification codes (i.e. participant 1–16). Data analyses followed golden standard methodology for analyzing qualitative data: quotes were labeled in the raw data and categorized in subsequent codes and concepts to come at a final model [25, 26]. Content analysis was conducted by two researchers (SB and GF) that independently examined the data. Open coding was used by SB and GF to independently examine the data for relevant quotes and group them together in codes. Subsequently, similar codes were grouped together into more general concepts by SB. Next, the codes and concepts were discussed between the two independent researchers (SB and GF) and themes were created and discussed. Last, interpretation of results and resulting themes were discussed with a third researcher, MH. Coding was done using qualitative software Atlas.ti to facilitate the labeling of quotes and the subsequent categorizing of these quotes into codes and concepts.

## Results

Three themes emerged from our focus groups: modification as the common practice, professional and patient factors influencing the decisions to adhere to or to modify protocols and organizational boundaries and flexibility as important contextual factors. Examples of concepts, codes and quotes related to the three themes can be found in Table 3.

**Table 3:** Examples of concepts, codes and codes for each theme

Concept(s)	Code (s)	*Quote*
Theme 1 Modification as the common practice		
Definition of personalizing treatments / Personalizing treatments	Intuition / How to personalize treatments: methods	*‘I think some therapists feel that they need to work according to their own insight and standards. And although this pattern seems less than a while ago, some therapists may still need a certain amount of freedom to do so’ …* ‘*for example: eclectic work, a bit of schema therapy, talking about modi, making a sociogram and talking about the past week’* (participant 15).
Definition of personalizing treatments / Knowledge about colleagues	Combine treatments from the start / Differences in conducting treatments	*‘I think that there are colleagues that forget the protocol and combine different techniques to form a protocol. The danger of this strategy is that this may lead to never ending treatments’* (participant 5).
Personalizing treatments	Do you personalize treatments?	*‘In patients with comorbid personality problems I will start with CBT, and if that does not work continue with schema therapy, and if there are topics I can use EMDR, I will also start EMDR, but not before discussing it with our team’* (participant 9).
Definition of personalizing treatments / Personalizing treatments	Flexible use within the protocol / Personalizing within CBT	‘*There is CBT, and then you personalize: what does this person need first from the protocol?... If somebody already has an active lifestyle, I will spend less time on behavioral activation compared to someone who has no activities’* (participant 6).
How are protocols being used? / Role of the organization	Opinions / Differences between organizations	*‘Both ways are not great, doing a lot of different interventions may lead to less focus, but it can be also very difficult to force people into a protocol’* (participant 4).
Knowledge about colleagues	Little knowledge	*‘I would like to see that every therapist uses the protocol, but I doubt whether this is the case in day-to-day practice’* (participant 10).
How are protocols being used?	Opinions	*‘That is why I like online or blended treatment, it helps you and the patient to really work through the protocol* (participant 2) *… while also leaving some space for topics that are not directly related to the protocol during the offline sessions’* (participant 14).
Theme 2 Professional and patient factors influencing the adherence to protocols		
How are protocols being used?	Therapist discipline	*’A therapist needs discipline to adhere to protocol, because there will always be the temptation to get stuck in a nice and friendly conversation about things that are not related to the primary goal of treatment’* (participant 11).
Solutions for better use of protocols	Experience and knowledge	*‘I believe it might be the way of thinking we need to change … If you change your mindset to ‘this intervention is helpful, or more effective’, you will make yourself, or aim to, organize the circumstances that are necessary to adapt the intervention’* (participant 15)*.*
Moderators of personalizing treatment protocols / Personalizing treatments	Patient factors / When to personalize?	*‘Some patients need more space, sometimes when I try to closely adhere to the protocol the patient will tell me his motivation is decreasing. In those cases, I had to stop strict adherence to the protocol*’ (participant 12).
Reasons to modify protocols	Patient factors	*‘For example, patients that experience crisis all the time, or when you just haven’t been able to use the protocol yet’* (participant 4).
Theme 3 Organizational boundaries and flexibility		
What is needed to adhere to treatment protocols?	Reports and evaluation of treatments and clear treatment structure	*‘Regular patient evaluations in the team help to structure the treatment for the therapist and the patient, while my colleagues help to focus on the treatment protocol and notice possible distractions from the treatment protocol’* (participant 15).
Role of the organization	A lot of freedom	*‘The advantage is that it is easier to do something you think is important, and sometimes that is very useful, at other times it will dilute your approach’* (participant 4).
Solutions for better use of protocols	Role of the manager and team	*‘It is important that the manager is motivated and has a clear rationale for what and why we are doing what we do’* (participant 9).
What is missing but necessary for adhering to protocols?	Having the same professional vision	*‘What I sometimes miss, is coming together and feeling supported by other colleagues. Having the same vision. This makes our job more difficult’* (participant 4)

Some quotes were incorporated into multiple codes and concepts

### Theme 1: Modification as the common practice

Although CBT and IPT protocols were known and used as a guidance by all participants, the therapists pictured a situation within their organizations where modification of protocols seemed to be common practice and happened in a range of different ways. On one extreme end of the range, participants mentioned that they thought treatment protocols are being ignored by some of their colleagues, who act on what they personally think is best for the patient. In contrast, on the other end of the range, participants mentioned the very strict adherence to a protocol. Between these two contrasts, participants mentioned four additional ways in which treatment protocols can be modified. For example, treatment protocols can be modified by combining different treatment protocols directly from the start to create an individualized treatment protocol adjusted to the patient. Examples are the combination of CBT protocols for different disorders, a combination of the CBT protocol with the protocol for eye movement desensitization reprocessing (EMDR) or combining the CBT protocol with protocols focused on sleep problems. It was mentioned that this method may lead to loss of focus. Another way to use treatment protocols was by starting with one protocol and switching to another protocol without finishing the protocol that was started first. In addition, it was mentioned that ingredients can be added or removed from treatment protocols. Examples mentioned were: CBT without behavioral activation, to add assertiveness training or running therapy to the CBT protocol or to spend one session on another intervention. Last, therapists mentioned flexible adherence to a protocol without losing the main lines of the protocol. Thus, mainly using one protocol while also making small adjustments, for example by giving more attention to some parts of the protocol.

Participants were ambivalent about these modifications. On the one hand, modifications were considered as unhelpful and not in line with the scientific evidence, which was considered the norm, whereas on the other hand strictly following protocols might interfere with the treatment process too. In addition, participants mentioned differences in modifications for different forms of psychotherapy: compared to IPT, CBT was experienced as a more structured treatment and therefore easier to adhere to.

### Theme 2: Professional and patient factors influencing the decision to adhere to or modify protocols

Participants described both professional- and patient-related factors that influenced the modification of treatment protocols. Factors related to the professional were: the type of training and supervision received, the years of work experience and some characteristics of the therapist as a person. Therapists trained in multiple therapeutic techniques will be more likely to try out different treatment techniques in the same treatment and modify treatment protocols quicker compared to therapists that were trained in a single treatment technique.

It was suggested that supervisors in the Netherlands follow and teach different approaches in terms of protocol adherence: whereas some supervisors seem to strictly adhere to protocols and use ratings scales to investigate which therapist skills were (not) used during the sessions, other supervisors seem less strict in protocol adherence. Not all supervisions use audio or video tapes to evaluate therapy sessions, which was suggested to increase adherence to protocols. Also, years of work experience seems to play a role in protocol adherence, with young psychologists having a stronger inclination to adhere to a protocol than more experienced therapists who might feel more flexibility to use and choose from different protocols. The use of e-health (i.e. structured online environments in which different sessions address different elements of the treatment protocol) was suggested to help to keep up with the protocol in case of distractions to topics that are not directly related to the treatment protocol but, according to the therapists, need to be addressed in the session (such as substantial life events or crisis).

A few personal characteristics of the therapists and patients were also considered relevant. For example, factors related to the patient were: being in a crisis, low cognitive abilities, the presence of suicidal behavior or suicidal intrusions, not accepting the protocol, low motivation for treatment, or the presence of comorbid problems (i.e. PTSD, personality disorders). There was some disagreement among the therapists about when to modify the adherence to the protocols, for example in the case of a higher session frequency. While some therapists who thought that two weekly sessions were unhelpful for patients with high levels of avoidance, other therapists believed that in case of avoidance, the first weekly session may help to tackle avoidance so that in the second session, after only a few days, one could entirely focus on the actual therapy content. In the same way, while some therapists mentioned that a higher session frequency may deepen therapy and enhance activation in severe depression, other therapists mentioned that this might just be too much for patients that are severely depressed. Beside these patient factors, therapists mentioned that it can be hard to accept that some patients do not improve, and that this may lead to longer treatments than is necessary or helpful for the patient. Also, being disciplined was considered important to prevent unnecessary distractions from the protocol, whereas therapists agreed that a change of mindset of the therapist is essential for successful implementation and adherence to treatment protocols.

### Theme 3: Organizational boundaries and flexibility

Participants indicated that a number of organizational factors strongly influence the way they adhere to or modify treatment protocols. These factors included: the attitude of the manager towards protocolised care, reorganizations, planning capacity and workspace, a coherent team vision on protocol implementation, or a main focus on production targets. Overall, limited planning capacity and workspace, a main focus on production targets and changes in the organization were mentioned to hinder the adherence to and implementation of treatment protocols. Combining these factors, three types of organizational cultures in terms of protocol implementation, appeared from the discussions with the therapists.

In the first type of mental health care location, therapists were confronted with standardized mental health care programs with hardly any options to change or adapt the treatment during the course of the treatment process. This helps to adhere to the protocol and to focus on one specific disorder, but may also be a disadvantage in the case the protocol does not fit the patient. For example, if during the first sessions it appears that the patient treated for depression is also suffering from comorbid post-traumatic stress disorder (PTSD), it would not be possible to apply PTSD techniques. The patient would first have to switch to another therapeutic team and overcome a waiting list period or finish the depression treatment before he or she can receive a treatment focused on PTSD.

In the second type of mental health care location, described in a very positive way by some of the therapists, there was some flexibility, but also a coherent shared vision and room for discussion within the team about which treatment protocol to use, and in what cases the therapist will have to deviate from this protocol*.* One of the locations that recently successfully switched from individualizing treatments to adherence to protocols as a standard, ingredients that were described for successful implementation were: an active, enthusiastic manager, enough supervision, team meetings and the possibility to try the new procedures before definitely implementing them.

In the third type of mental health care location, therapists experienced a lot of flexibility and limited social control by the team. In these organizations, therapists seemed less bothered by the organizational barriers, organize their own agenda and seemed able to decide independently of their manager whether or not to apply a new protocol, such as a higher session frequency. However, this amount of flexibility was also linked to the risk of less focused treatment*.*

## Discussion

In the context of a randomized trial, we investigated the perspective of fifteen therapists on the adherence to CBT and IPT protocols in specialized mental health care in the Netherlands. Modification and deviation from protocols by therapists was viewed as common practice within the organizations involved in the study. We identified a range of ways in which treatment protocols were modified and different professional and patient factors that influenced the decisions of therapists to divert from the protocol. In addition, adherence to protocols was strongly related to the organizational context, especially to the flexibility offered to therapists to choose and modify treatment protocols. The organizations involved in the study varied from a very inflexible culture to a very flexible culture in terms of adherence to protocols.

Modifications from treatment protocols described by the interviewed therapists were partly in line with current research evidence or clinical guidelines. For example, acting on what you personally think might be best or combining protocols from the start of treatment is not supported by any research or guidelines [12, 27]. On the other hand, therapists also mentioned to adapt treatment protocols by making modifications that seem to be fidelity consistent. This method seems an excellent example of how to modify treatment protocols without losing focus or guidelines [17] and is in line with a recent study that showed that flexibility of the use of treatment techniques was related to better treatment outcomes [28]. Other modifications mentioned, such as switching to another protocol, or adding or removing elements from the intervention were less straightforwardly linked to research outcomes and guidelines and might even be seen as fidelity-inconsistent [16]. Beside treatment modifications, therapists’ suggestions concerning patient characteristics that influence their decisions to adhere to protocol were consistent with the literature. For example, research and guidelines show that trauma should be treated before the depression [29], and that in the case of complex depression (i.e. comorbid problems, previous inadequate response), treatment for a comorbid personality disorder should be considered as one of the first choices [27]. In addition, studies have come up with adaptions to treatment protocols to deal with suicidal problems [30, 31] or to target problems with memory during CBT [32].

Therapists pointed to multiple factors that can help to decide when to modify protocols and how to decrease the undesirable lack of adherence to protocols. Suggestions were in line with studies showing that training, supervision or the years of work experience of the therapist are related to better delivery of treatment [20, 33–36], feedback, observation and consultation improve the adoption and retention of innovations [34] and that regular meetings with colleagues may increase the use of research findings [37, 38]. Besides, therapists mentioned that social pressure, for example by use of consistent patient evaluations or observational measures such as video-tapes of sessions, might be an important factor that would help to increase adherence to protocol. This is in line with other studies that suggested that the use of observational techniques, such as video material, is essential for the adequate reflection and feedback [39]. Nevertheless, a recent pilot study indicated that compared to social rewards (i.e. defined as being publicly recognized for adherent CBT delivery), financial rewards (i.e. $100 if a session that was selected and watched in the context of the study met criterion for adherent CBT delivery) may even lead to better increase of adherence to treatment protocols [40].

The present study has several strengths and limitations. A strength of the present study was that it was the first to investigate the adherence to and modifications of CBT and IPT protocols and its relation with professional, patient and organizational factors in the Dutch specialized mental health care for depression from a therapist viewpoint. The focus group method enabled the discussion of protocol fidelity issues in depth with clinicians. Also, a large variation of therapists and organizations participated and by presenting issues that came up in the first focus group to the participants in the second group, the current study was able to confirm the themes and enrich the data. The study led to clear recommendations for therapists and organizations to improve the adherence to treatment protocols in Dutch specialized mental health care. A limitation of the study was a lack of representation from all organizations participating in the original trial. Also, focus groups generate data on what clinician’s say they do, but this might not reflect actual behavior in daily practice. Another limitation was that the majority of the clinicians involved in the focus group were still in training. Although there is yet little literature on the relation between therapist experience and protocol adherence, it is possible that less experienced therapists have different views on protocols adherence in clinical practice compared to more experienced therapists. Finally, the selection of therapists may have led to selection bias as it might be possible that the therapists who volunteered to participate in the focus groups might be more amenable to protocol-based treatments.

These findings lead to several recommendations for therapists and organizations. First, protocol use and adherence in clinical practice can be increased by distinguishing between types of modification and relating these changes to clinical guidelines. Second, use of observational material, such as video or audio tapes of treatment sessions, may improve treatment evaluations. Third, in regard to the organization, time and space for supervision and regular team meetings should be created. Regular team meetings may not only help to increase adherence to protocol (i.e. for example by enabling discussion of patient characteristics that make it difficult to adhere to protocol, decreasing unnecessary distractions due to other interventions or helping to prevent the continuation of never-ending treatments), but also create room for discussion about what factors should lead to a modification of the protocol and increase a shared treatment vision. As therapists indicated that more social control will lead to better treatments, organizations should implement regular patient evaluations and monitor not only the quality of treatment, but also that of supervision. Managers may play an important role in increasing a positive environment that enables discussion about the adherence to treatment protocols and could play a role in monitoring what modifications are in line with the scientific literature and the team vision. In addition, piloting of a new intervention before definite implementation (for example: testing the intervention by a small group of employees) may help to increase support of the intervention and adherence to its protocol. Recommendations for clinical practice are summarized in Table 4.

**Table 4:** Recommendations for clinical practice to increase adherence and a shared vision on treatment protocols

Recommendations for clinical practice	
Therapists	
• Distinguish between ways of modifying treatment protocols and relate these modifications to clinical guidelines	
• Use observational material (i.e. video/audio) during supervision	
• Discuss treatment modifications with colleagues	
• Plan regular patient evaluations	
Mental health centers	
• Create time and space for supervision of treatments and monitor it’s quality	
• Organize regular team meetings for discussions about protocol modifications	
• Recognize and define the role of the manager in relation to protocol implementation	
• Pilot protocols for new interventions or methods	

The present study led to multiple hypotheses about what common protocol deviations are and what drives them that should be addressed in future studies to help therapists make informed choice about how and when to modify treatment protocols and enhance evidence-based practice. First, further research into the adherence to protocols for specific patient characteristics, such as comorbid disorders, cognitive abilities, patient motivation for treatment or suicidality, is needed. Studies should not only investigate the effects of additional interventions or techniques, but also specify how these specific patient characteristics can be treated within the protocol and, when therapists should modify or switch to another protocol. The study of Beidas and colleagues [41] provides a good example of how to provide a manual that discusses the implementation of a flexible manualized treatment protocol without losing fidelity. Their study presents an elaborate discussion (including video material) of how their specific treatment protocol can be used in a flexible way for different challenges that might be encountered when using the protocol, such as social skill deficits or depressive symptoms, and may serve as an example for future studies on flexible adherence to treatment protocols for depression. In addition, it is possible that protocol modifications will be less needed if we can find out what treatment or protocol works for whom. Recent studies already showed that treatment outcomes can be improved by using patient characteristics to predict from which one of two treatments the individual patient benefits most [42, 43] and future studies should investigate how these predictions can be applied in a clinical setting. Second, future studies should focus on the effects of flexible adherence to CBT and IPT protocols on treatment outcomes and should investigate whether different forms of protocol use will lead to different treatment outcomes. Third, the role of team influence and supervision (i.e. the frequency, structure, and methods of supervision) in the adherence to treatment protocols, quality of treatment and subsequent treatment outcome should be investigated [36, 44]. Following a recent study, future studies should investigate whether and how standardized assessments can be used improve training and supervision in the adherence to treatment protocols [45].

## Conclusions

According to therapists, treatment protocols for CBT and IPT in Dutch specialized mental healthcare are modified in multiple ways and not all of these modifications are in line with existing research evidence or guidelines. Differences between therapists in how treatment protocols are used and modified are related to professional and patient characteristics and to the flexibility offered by the organizational context. Regular supervision, team meetings and a shared vision were identified as potentially crucial factors to increase adherence to treatment protocols, while additional organizational factors, among which a change in mindset, may facilitate adequate implementation. Gaps in current research evidence should be addressed to help therapists make an informed choice about how and when to modify treatment protocols and enhance evidence-based practice.

## Abbreviations

CBT: Cognitive behavioral therapy
EMDR: Eye movement desensitization reprocessing
IPT: Interpersonal therapy
PTSD: Posttraumatic stress disorder
RCT: Randomized controlled trial
